# The Role of Surgery in Small-Cell Lung Cancer in the Chemoimmunotherapy Era

**DOI:** 10.3390/cancers18213484

**Published:** 2026-10-29

**Authors:** Francesco Petrella, Andrea Cara, Enrico Mario Cassina, Jessica Dell’Agosto, Lidia Libretti, Emanuele Pirondini, Federico Raveglia, Antonio Tuoro

**Affiliations:** 1 Department of Thoracic Surgery, Fondazione IRCCS San Gerardo dei Tintori, 20900 Monza, Italy; andrea.cara@irccs-sangerardo.it (A.C.); enricomario.cassina@irccs-sangerardo.it (E.M.C.); jessica.dellagosto@unimi.it (J.D.); lidia.libretti@irccs-sangerardo.it (L.L.); emanuele.pirondini@irccs-sangerardo.it (E.P.); federico.raveglia@irccs-sangerardo.it (F.R.); antonio.tuoro@irccs-sangerardo.it (A.T.); 2 Department of Oncology and Hemato-Oncology, University of Milan, Via Festa del Perdono 7, 20122 Milan, Italy

**Keywords:** small-cell lung cancer, surgery, immunotherapy, durvalumab, molecular subtypes

## Abstract

The modern role of surgery in small-cell lung cancer (SCLC) is best understood as an unresolved question rather than a settled recommendation. Older randomized trials established that surgery has no role in unselected SCLC, but these trials predate PET-CT staging, endosonographic mediastinal staging, and immunotherapy, and therefore cannot answer today’s clinical question in modern, invasively staged, node-negative disease. Current guideline support for resection in cT1–2N0 SCLC rests largely on retrospective, registry-based data (SEER, NCDB) that carry substantial selection bias rather than randomized confirmation. Meanwhile, the nonsurgical comparator has advanced: chemo-radiotherapy plus durvalumab consolidation, validated in the ADRIATIC trial, now defines the modern limited-stage standard against which any surgical strategy must be benchmarked. Molecular subtyping (ASCL1, NEUROD1, POU2F3, inflamed phenotypes; TP53/RB1 co-alteration) and emerging neoadjuvant and perioperative chemo-immunotherapy data introduce plausible, though unproven, avenues for patient selection and treatment sequencing. Surgical tissue also offers a translational platform for studying immune microenvironment, clonal evolution, and treatment resistance. This review argues that surgery for SCLC is neither obsolete nor an established standard in isolation. Its future role requires two distinct lines of prospective investigation: comparisons of upfront surgery with neoadjuvant chemoimmunotherapy followed by resection, which address treatment sequencing, and comparisons of surgery-based multimodality treatment with definitive concurrent chemoradiotherapy followed by durvalumab, which address the incremental value of surgery over contemporary nonsurgical care.

## Simple Summary

Historical randomized trials showed no benefit from surgery in unselected SCLC, but those trials predate modern PET/CT and invasive mediastinal staging, molecular subtyping, and immunotherapy, so they cannot directly answer the question for today’s carefully staged, very early (cT1–2N0) disease. Current support for resection in cT1–2N0 SCLC comes mainly from retrospective, registry-based data with significant selection bias, not prospective randomized confirmation. NCCN guidelines allow for lobectomy with mediastinal lymph node dissection in this narrow, well-staged subgroup, but nodal upstaging changes management substantially. The nonsurgical comparator has also advanced: chemoradiotherapy plus consolidation durvalumab (ADRIATIC) is now the modern limited-stage standard, and any surgical strategy must be benchmarked against this regimen rather than older chemoradiotherapy alone. Molecular heterogeneity (ASCL1, NEUROD1, POU2F3, inflamed subtypes; TP53/RB1 co-alteration) may eventually help select patients most likely to benefit from local therapy, and early neoadjuvant/perioperative chemoimmunotherapy series show encouraging pathologic response rates, though data remain immature. Surgery is neither a default treatment for early-stage SCLC nor an obsolete approach; it remains an option for carefully selected, thoroughly staged cT1–2N0 patients within a multidisciplinary framework.

## 1. Introduction

The role of surgery in small-cell lung cancer (SCLC, or “microcitoma”) is undergoing genuine reconsideration after decades of near-categorical exclusion. That exclusion traces to two phase III trials from the 1970s and 1990s, which found no survival benefit for surgery over chemotherapy/radiotherapy and led international guidelines to largely discourage resection [1,2]. These trials, however, enrolled few patients who would meet modern definitions of node-negative T1-2N0 disease and predate current staging technology, including FDG-PET/CT, EBUS-guided mediastinal staging, and low-dose CT screening [3,4]. Today, improved imaging and lung cancer screening have made true clinical stage I SCLC an identifiable, if rare (<5% of cases), entity. Contemporary retrospective and population-based cohorts—including NCDB analyses—consistently show a survival advantage for surgical resection (particularly lobectomy with mediastinal lymphadenectomy) plus adjuvant systemic therapy compared with chemo-radiation alone in this subgroup, with 5-year overall survival approaching 47–58% versus roughly 30% [5,6]. This creates a paradox: the old randomized evidence says no, while newer, better-staged observational evidence says yes, and no modern randomized trial has re-tested surgery against current standards of staging and systemic therapy, leaving selection bias as an unresolved confounder. Importantly, two distinct uncertainties remain: whether surgery should be integrated with systemic therapy using an upfront or neoadjuvant sequence, and whether a surgery-based multimodality strategy offers an incremental benefit over definitive chemoradiotherapy followed by durvalumab in eligible patients. These questions require different study designs and should not be addressed interchangeably.

Compounding this uncertainty, the therapeutic landscape has been transformed by the arrival of immune checkpoint inhibitors, which have improved outcomes in both extensive-stage disease (IMpower133 [7,8], CASPIAN [9]) and, more recently, limited-stage disease with durvalumab consolidation. Whether immunotherapy should now be integrated into multimodality treatment of surgically resected early-stage SCLC remains undefined. The new clinical question, therefore, is no longer whether surgery “works” in SCLC as historically studied, but whether carefully staged, node-negative patients—managed within a multidisciplinary framework incorporating modern staging, lobectomy, and adjuvant systemic therapy with or without immunotherapy—represent a distinct population in whom local therapy is warranted. Two distinct research questions must be addressed: whether different treatment sequences improve outcomes among patients receiving surgery, and whether a surgery-based multimodality strategy provides additional benefit over definitive concurrent chemoradiotherapy followed by durvalumab in eligible patients. The former evaluates treatment sequencing, whereas the latter addresses the comparative value of surgery itself. Given the rarity of surgically eligible SCLC, a definitive randomized comparison may be difficult to conduct. International prospective registries, cooperative-group studies, platform-based approaches, and target-trial emulation may therefore complement randomized evidence. Until comparative data become available, the benefit of surgery over contemporary nonsurgical treatment remains unproven [10].

This review examines the evolving role of surgery in SCLC, synthesizing historical perspectives, contemporary evidence, current guideline recommendations, and emerging trends in surgical management. By critically evaluating the available data, this review aims to provide a comprehensive framework for understanding when and how surgery should be incorporated into the treatment of this challenging malignancy, while acknowledging the need for prospective validation of current practice patterns.

## 2. Methods

This structured narrative review evaluated contemporary evidence on the role of surgery in SCLC, with particular emphasis on early-stage disease and the integration of surgery with systemic therapy, radiotherapy, and immunotherapy.

A literature search was performed in PubMed/MEDLINE and Google Scholar, covering publications from January 2013 through August 2026. Search terms included “small cell lung cancer,” “SCLC,” “surgical resection,” “lobectomy,” “multimodality treatment,” “adjuvant chemotherapy,” “immunotherapy,” “chemo-immunotherapy,” “early-stage disease,” “limited-stage disease,” “molecular subtypes,” and “neoadjuvant therapy.” Reference lists of key publications and guidelines were also screened for additional relevant studies.

Clinical trials, cohort studies, case–control studies, systematic reviews, meta-analyses, guidelines, and relevant translational studies published in English were considered. Titles and abstracts were screened for relevance, followed by full-text assessment of potentially eligible publications. Given the heterogeneous nature of the available evidence, findings were synthesized qualitatively rather than quantitatively.

Study limitations and potential sources of bias, particularly selection bias and residual confounding in retrospective studies, were considered when interpreting the evidence. No formal risk-of-bias tool or evidence-grading system was applied because this article was designed as a structured narrative review rather than a formal systematic review or meta-analysis.

## 3. Current Evidence for Surgery

Current evidence for surgery in SCLC derives almost entirely from retrospective, population-based cohorts rather than modern randomized trials, and it converges on a benefit confined largely to node-negative disease. For cT1-2N0M0 disease, the largest propensity-matched NCDB analysis (n = 1002) showed 5-year overall survival of 47.6% with surgery plus adjuvant chemotherapy versus 29.8% with concurrent chemo-radiation, a benefit preserved even after restricting to comorbidity-free patients [7]. In the SEER analysis by Yu et al., 5-year OS after lobectomy was 50.3% without radiotherapy and 57.1% with radiotherapy [11]. However, all of this evidence shares the same structural limitation: patients selected for surgery are inherently healthier, have smaller tumors, and undergo more rigorous invasive mediastinal staging than those managed nonoperatively, and even sophisticated propensity-score matching cannot fully correct for this residual selection bias, which likely inflates the observed benefit.

Regarding extent of resection, lobectomy is preferred: a meta-analysis of 41,483 patients found surgery-based treatment improved OS over nonsurgical treatment only in retrospective data and not in the two older RCTs, while sublobar resection was inferior to lobectomy [9]. More recent SEER and NCDB analyses, however, show inconsistent findings after adjustment—some [4,12] found the lobectomy advantage disappeared after propensity matching, suggesting sublobar resection may be non-inferior in carefully matched stage I cohorts, while others [13] continued to show sublobar resection independently associated with worse survival. This inconsistency likely reflects small sample sizes and unmeasured confounding (comorbidity, pulmonary reserve) rather than true oncologic equivalence.

The controversy in SCLC remains unresolved: surgery is reserved for carefully staged, node-negative patients, but no randomized trial directly compares lobectomy with sublobar resection in SCLC, so current practice is largely extrapolated from non-small-cell lung cancer (NSCLC). Lobectomy remains the oncologically safer option for physiologically fit patients because it maximizes parenchymal margins and nodal assessment. Conversely, anatomic segmentectomy may be reasonable in selected elderly or physiologically compromised patients when lobectomy would carry unacceptable functional or perioperative risk; however, evidence that it meaningfully preserves function in patients with severely impaired cardiopulmonary reserve remains limited. Modern NSCLC trials support sublobar resection mainly for peripheral, ≤2 cm, pathologically node-negative tumors, and some demonstrate higher locoregional recurrence after sublobar surgery, limiting direct applicability to the more aggressive biology of SCLC. Thus, if resection is pursued, segmentectomy with rigorous mediastinal staging and adequate margins is generally more defensible than wedge resection; otherwise, definitive nonsurgical treatment may be preferable to compromising oncologic clearance.

Nodal disease is the area of greatest inconsistency across the literature. Some SEER/NCDB analyses report a survival benefit from surgery extending even to N1 disease (5-year OS 31.4% vs. 26.3% with chemoradiation) and select stage III/N2 cohorts, while NCCN restricts surgical candidacy to cN0 disease confirmed by invasive mediastinal staging, reserving N1/N2 findings discovered pathologically for adjuvant radiation rather than a primary surgical indication [6,14,15]. This reflects a real distinction: retrospective series often report outcomes by pathologic nodal status, which is subject to survivor and selection bias; on the other hand, NCCN’s guidance is built on preoperative staging as the actual decision point. cN0 by invasive staging is required before offering surgery, and unexpected N1/N2 found at resection is managed with adjuvant therapy rather than treated as evidence that surgery should be offered upfront for known N1/N2 disease [6].

Complete staging is essential before considering surgery for presumed early-stage SCLC. The preoperative work-up should include contrast-enhanced CT of the chest and abdomen, FDG-PET/CT for assessment of loco-regional and distant disease, and brain MRI to exclude intracranial metastases. Brain imaging is particularly important because apparently localized disease may be upstaged by occult CNS involvement. Invasive mediastinal staging is required when surgery is contemplated. EBUS-guided transbronchial needle aspiration (EBUS-TBNA), supplemented by endoscopic ultrasound-guided sampling when appropriate, is a minimally invasive first-line approach for accessible mediastinal lymph nodes. If endosonographic sampling is negative or nondiagnostic despite persistent clinical or radiological suspicion, confirmatory surgical staging, usually by mediastinoscopy or another station-appropriate surgical approach, should be considered. Importantly, mediastinal staging and assessment of hilar/interlobar N1 disease are distinct, although complementary, components of nodal evaluation. Mediastinal N2/N3 involvement may preclude a surgery-based strategy, whereas hilar and intrapulmonary N1 involvement should be assessed through appropriate imaging, endosonographic sampling when feasible, and systematic intraoperative nodal evaluation. A clinically node-negative mediastinum does not, by itself, exclude N1 disease. Accordingly, surgery should be considered only after comprehensive staging and multidisciplinary review [3,4].

Mediastinal staging in presumed early-stage SCLC is challenging because occult nodal disease may be missed on CT or PET, yet mediastinal involvement can redirect a patient away from a surgical pathway. Endobronchial ultrasound-guided transbronchial needle aspiration (EBUS-TBNA) is generally preferred when accessible because it is less invasive than mediastinoscopy and can combine diagnosis with nodal staging; however, its yield is operator- and station-dependent, and some stations—particularly 5, 6, 8, and 9—may require endoscopic ultrasound or another approach. A negative or non-diagnostic EBUS should therefore not automatically permit resection when imaging or tumor characteristics maintain high suspicion; mediastinoscopy remains appropriate for confirmatory staging in selected cases, despite the added anesthesia, morbidity, and delay. This is clinically important because guidelines emphasize excluding mediastinal nodal involvement whenever surgery is being considered for limited-stage SCLC, while occult N2/N3 disease may make nonsurgical multimodality treatment more appropriate.

All patients with resected SCLC should receive adjuvant platinum–etoposide chemotherapy, provided they are medically fit. Postoperative radiotherapy (PORT) should be determined by pathological nodal status, resection margins, and multidisciplinary assessment rather than applied uniformly to all node-positive patients. For completely resected pN0 disease, adjuvant chemotherapy without routine PORT is appropriate. For unexpected pN1 disease involving hilar, interlobar, or intrapulmonary nodes, adjuvant chemotherapy is indicated, while PORT should be considered selectively according to the extent and distribution of nodal involvement, margin status, and individual locoregional risk; evidence supporting routine PORT in pN1 SCLC remains limited. In contrast, unexpected mediastinal pN2 disease warrants adjuvant chemotherapy combined with mediastinal radiotherapy, preferably concurrently when clinically feasible. PORT is also recommended for consideration in R1/R2 resections [3,4].

The overall limits of current evidence—absence of contemporary RCTs, database heterogeneity, and inability to fully adjust for selection—mean that surgery should be considered only within multidisciplinary, guideline-concordant algorithms rather than generalized to all node-positive presentations (Table 1).

## 4. Non-Surgical Benchmark in SCLC

Consolidation durvalumab after concurrent chemoradiotherapy has redefined the nonsurgical benchmark against which any future surgical strategy for SCLC must now be measured. For three decades, the standard of care for limited-stage SCLC remained concurrent platinum-etoposide chemoradiotherapy, yielding a median overall survival of only 25–30 months and a 5-year survival of 29–34%, with essentially no meaningful systemic therapy advances despite successive trials testing radiotherapy fractionation and dose intensification [16]. This stagnancy ended with the phase 3 ADRIATIC trial, which randomized patients with limited-stage SCLC without progression after chemo-radiotherapy to consolidation durvalumab versus placebo. Durvalumab produced a striking improvement in median overall survival (55.9 vs. 33.4 months) and progression-free survival (16.6 vs. 9.2 months), representing the first major systemic therapy advance in this disease in three decades [13]. This benefit has proven consistent across chemotherapy backbone, radiotherapy fractionation schedule, and use of prophylactic cranial irradiation, and it has not come at the cost of quality of life [17,18]. Consolidation durvalumab is now incorporated as a category 1 recommendation in the NCCN algorithm for limited-stage disease following response assessment [6]. This development fundamentally changes the comparator against which surgery must be judged. Historical randomized trials and retrospective surgical series were conducted against a chemoradiotherapy backbone with a ceiling of roughly 30 months’ median survival; any survival advantage attributed to surgery in that era must now be reinterpreted against a nonsurgical benchmark exceeding 55 months. Consequently, surgery for very early-stage SCLC can no longer be justified, or dismissed, based on comparisons with outdated chemoradiotherapy-alone cohorts. Any contemporary case for resection in cT1-2N0 disease must demonstrate an incremental benefit over chemoradiotherapy plus durvalumab, not over historical controls, reframing the central question of ongoing and future surgical trials in SCLC.

## 5. The Role of Immunotherapy in SCLC

The paradox of small-cell lung cancer immunotherapy—a tumor with among the highest tumor mutational burdens (TMB) of any solid malignancy yet only modest clinical benefit from checkpoint blockade—reflects multiple convergent, tumor-intrinsic and microenvironmental mechanisms of immune evasion. Despite high TMB predicting robust neoantigen generation and, correspondingly, strong T-cell responses in some patients (notably those with paraneoplastic neurological syndromes), checkpoint blockade produces durable responses in only ~15% of unselected patients, and TMB itself has failed as a reliable predictive biomarker across prospective analyses of IMpower133 [7,8], CASPIAN [9], and KEYNOTE-604 [1,5]. A central mechanism is downregulation of MHC class I antigen presentation machinery, occurring in up to ~85% of SCLC cases, mediated by epigenetic silencing (notably EZH2/PRC2-driven repression of HLA-I genes), loss of β2-microglobulin, and oncogenic signaling such as ERBB2 activation—all of which blunt CD8+ T-cell recognition and promote metastatic spread [1,19,20,21]. Compounding this, SCLC tumors exhibit a “cold” and markedly heterogeneous T-cell receptor repertoire, with T-cell exclusion linked to high copy-number aberration burden and loss of IFN-γ pathway genes secondary to combined TP53/RB1 inactivation [22]. The tumor microenvironment is further dominated by myeloid immunosuppression—tumor-associated macrophages, myeloid-derived suppressor cells, and regulatory T cells—along with CXCR4+ neutrophils and S100A8+ M2-polarized monocytes that actively exclude cytotoxic T cells, producing an “immune-desert” phenotype [23,24]. Neuroendocrine phenotype also shapes immunogenicity: transcription factor-defined subtypes (SCLC-A/ASCL1, SCLC-N/NEUROD1, SCLC-P/POU2F3, SCLC-I/inflamed) show distinct immune landscapes, with neuroendocrine-dominant ASCL1/NEUROD1 tumors typically non-inflamed and MHC-I-low, while the inflamed subtype (and neuroendocrine-inflamed tumors specifically) show greater T-cell infiltration and appear to derive the most benefit from anti-PD-L1 therapy [25]. Finally, PD-L1 expression itself is infrequently detected on SCLC tumor cells (as low as 0–17% in various cohorts) and has not correlated with response in randomized trials, leaving oncologists without a validated biomarker to select patients—a limitation that, together with TMB’s inconsistent predictive value, underscores why current immunotherapy benefit in SCLC remains population-level rather than individually predictable [26].

The role of perioperative immunotherapy in resectable SCLC remains investigational. Although durvalumab has an established role in extensive-stage SCLC following response to platinum-based chemotherapy, this evidence should not be extrapolated to the adjuvant or perioperative setting after surgery. The efficacy, safety, optimal timing, and duration of durvalumab in resectable SCLC have not been established. Its use in this setting should therefore be limited to appropriately designed clinical trials, rather than considered standard postoperative care.

## 6. SCLC Molecular Heterogeneity and Its Implications for Surgery

SCLC’s near-universal TP53/RB1 co-inactivation was long thought to produce a biologically uniform disease, but transcription factor-defined molecular subtyping now reveals substantial heterogeneity with potential implications for selecting surgical candidates. TP53 and RB1 loss represents the essential initiating event, occurring in roughly 92% and 74% of tumors, respectively, and drives downstream lineage plasticity through SOX2 upregulation and REST-mediated dedifferentiation [21].

Superimposed on this shared genomic backbone, differential expression of lineage transcription factors defines four principal subtypes: SCLC-A (ASCL1-driven, the classic neuroendocrine-high phenotype and most common, comprising 42–70% of cases), SCLC-N (NEUROD1-driven, a variant neuroendocrine phenotype often arising from SCLC-A via MYC-driven transition), SCLC-P (POU2F3-driven, a non-neuroendocrine tuft-cell-like phenotype), and SCLC-I (an inflamed, non-neuroendocrine phe notype with high immune-gene expression that derives the greatest bene fit from checkpoint blockade) [27]. Critically, these subtypes are not fixed: intratumoral heterogeneity and dynamic subtype switching—particularly transition from ASCL1-high to NEUROD1-high to YAP1-high/inflamed states—occur under therapeutic pressure and are implicated in acquired platinum and immunotherapy resistance [28].

In surgically resected cohorts specifically, subtype has shown prognostic associations: an international multicenter study of 386 resected specimens found high ASCL1 expression to be an independent negative prognosticator, while high POU2F3 expression correlated with improved survival, and no distinct YAP1-defined subtype was identified by immunohistochemistry [29]. A separate whole-section analysis of 73 resected tumors similarly found YAP1 positivity to be an independent poor prognostic factor after surgery, with increased mediastinal nodal recurrence [30]. A smaller resectable-SCLC cohort found SCLC-P to be an independent poor prognostic factor by multivariate analysis, without correlation between subtype and PD-L1 expression or CD8+ tumor-infiltrating lymphocytes [31]. These retrospective, hypothesis-generating data suggest that neuroendocrine-inflamed or immune-enriched phenotypes might eventually help identify patients most likely to benefit from surgery, or from surgery combined with adjuvant immunotherapy, but no subtype has been prospectively validated to guide surgical selection, and current NCCN eligibility for resection is based solely on clinical/pathologic stage (T1–2N0), not molecular phenotype [6].

## 7. Chemo-Immunotherapy and the Emerging Perioperative Paradigm

Chemoimmunotherapy has become the established standard for extensive-stage SCLC and, more recently, for limited-stage consolidation, while neoadjuvant and perioperative applications around surgery remain investigational. In extensive-stage disease, adding a PD-L1 inhibitor to platinum-etoposide is a category 1, guideline-endorsed standard: IMpower133 (atezolizumab) [7,8] showed a median OS of 12.3 vs 10.3 months and CASPIAN (durvalumab) [9,32] showed 12.9–13.0 vs. 10.3–10.5 months, with subsequent negative or marginal trials (KEYNOTE-604, tremelimumab-containing CASPIAN arm) underscoring that benefit is modest and agent-specific rather than a uniform class effect In limited-stage disease, established evidence supports immunotherapy only as consolidation after chemoradiotherapy (ADRIATIC, durvalumab) [13], whereas concurrent administration with radiotherapy (NRG/Alliance LU005, atezolizumab) [33] failed to improve OS, and neoadjuvant/induction chemoimmunotherapy prior to surgery remains experimental.

The rationale for extending chemoimmunotherapy into the perioperative space for very early-stage SCLC draws on the NSCLC paradigm, where neoadjuvant nivolumab plus chemotherapy (CheckMate 816) [34] improved pathologic complete response and event-free survival, and more recently overall survival, and where perioperative durvalumab (AEGEAN) [35] similarly improved pathologic complete response (pCR) and event-free survival (EFS) [36,37].

Direct SCLC data are limited to small, mostly retrospective or single-arm series. Several Chinese cohorts report that adding a PD-(L)1 inhibitor to neoadjuvant chemotherapy increases pathologic complete response (up to 35.7–50.0%) and major pathologic response (53.6–72.7%) compared with chemotherapy alone, with acceptable toxicity and, in some series, improved surgical resection rates and survival trends [36,37,38,39]. The phase 2 LungMate-005 trial (mostly stage III patients) reported a 92.5% objective response rate with induction anti-PD-L1 plus chemotherapy, with 61.9% major pathologic response (MPR) and 42.9% pCR among the subset who proceeded to surgery [40]. Postoperative (adjuvant) immunotherapy after resection is not addressed by prospective SCLC data; current NCCN guidance for resected disease specifies postoperative systemic chemotherapy (with mediastinal RT for N2/N3 disease) rather than adjuvant checkpoint inhibition, distinct from the durvalumab consolidation pathway reserved for chemoradiotherapy-treated, unresected limited-stage disease [6].

In summary, what is established is chemoimmunotherapy for extensive-stage disease and durvalumab consolidation after definitive chemoradiotherapy for limited-stage disease; what remains experimental is any neoadjuvant, perioperative, or postoperative immunotherapy strategy built around surgical resection, which currently rests on small retrospective cohorts and single-arm phase 2 data rather than randomized evidence.

## 8. Surgery as Translational Platform in SCLC Therapy

Because surgical resection occurs in fewer than 5% of SCLC cases, resected tissue represents a uniquely underexploited translational resource whose scarcity has historically constrained mechanistic research in this disease. Unlike advanced-stage biopsies, which are typically small, crush-artifact-laden fine-needle aspirates yielding little material for detailed biochemical study, surgical specimens provide sufficient tumor bulk—paired with adjacent normal lung—for integrated genomic, transcriptomic, proteomic, and epigenomic profiling, as demonstrated by proteogenomic analyses of resected treatment-naive tumors [40]. This tissue enables refinement of transcription factor-defined molecular subtyping (ASCL1, NEUROD1, POU2F3, and inflamed/YAP1-high phenotypes) directly in early-stage, surgically curable disease, which is biologically distinct from the metastatic samples that dominate existing genomic databases [41]. Resected specimens also permit spatially resolved characterization of the immune microenvironment, including tumor-infiltrating lymphocyte density, PD-L1 expression, and macrophage-epithelial interactions; spatial transcriptomic work has shown that distinct epithelial cell states and macrophage “domestication” programs correlate with immunochemotherapy response versus resistance, findings that could eventually stratify surgical candidates for perioperative immunotherapy [42]. Beyond static tissue analysis, surgery creates a platform for longitudinal circulating biomarker studies. Postoperative ctDNA is an independent predictor of recurrence in resectable lung cancer, detectable a median of 6.6 months before radiologic relapse, and tumor-informed assays derived from resected tissue can enable more sensitive minimal residual disease (MRD) monitoring than in unresectable disease, where tumor-informed panels are unavailable [42]. CLC-specific ctDNA studies demonstrate that dynamic cell-free DNA kinetics track chemotherapy and immunotherapy response and can reveal “recrudescence” patterns marking emerging resistance [43]. Paired pre- and post-treatment surgical or autopsy samples have also illuminated clonal evolution, showing that SCLC frequently follows branched phylogenies from a dominant ancestral clone, with platinum chemotherapy inducing subclonal diversification and ancestral clones re-emerging under subsequent therapy [44]. Collectively, surgical tissue and its associated circulating biomarkers offer an important, currently underused window into subtype plasticity, immune microenvironment dynamics, and mechanisms of chemoresistance that could ultimately inform patient selection for surgery-based, multimodal strategies—though this remains a research opportunity rather than an established clinical application.

## 9. Future Scenarios for Surgical Treatment of SCLC

A rational future trial framework for surgery in SCLC must specify patient selection, biological stratification, treatment sequencing, systemic and radiotherapy backbone, central nervous system (CNS) strategy, and translational endpoints, rather than testing surgery as an undifferentiated intervention.

Which patients: Trials should enroll only patients with pathologically confirmed, invasively staged node-negative disease (clinical stage I–IIA, T1–2N0M0) after mediastinoscopy or endosonographic staging, since this is the population NCCN identifies as most likely to benefit from resection and in whom occult nodal disease must be excluded before committing to a surgery-based strategy. Selected T3N0 or N1 patients could be considered in exploratory cohorts but should be analyzed separately given their distinct prognosis and radiation implications [6].

Which molecular subtype: Prospective trials should incorporate ASCL1/NEUROD1/POU2F3/inflamed subtyping and TP53/RB1 status at enrollment, testing prospectively whether inflamed or antigen-presenting-competent phenotypes preferentially benefit from surgery-based, immunotherapy-containing strategies, since current subtype data derive almost entirely from advanced-stage or retrospective surgical cohorts.

Treatment sequencing within surgery-based strategies: In carefully selected patients with invasively staged cT1–2N0M0 SCLC who are eligible for resection, a randomized comparison of upfront lobectomy with systematic mediastinal lymph node dissection followed by adjuvant treatment versus neoadjuvant chemoimmunotherapy followed by surgery could evaluate treatment sequencing. Relevant endpoints would include event-free survival, overall survival, pathological response, treatment completion, perioperative morbidity, and time to initiation and completion of postoperative therapy. Such a study would determine whether the timing and integration of systemic therapy improve outcomes among patients selected for surgery; it would not establish whether surgery itself is superior to nonsurgical treatment.

Surgery-based multimodality treatment versus contemporary nonsurgical treatment: The more fundamental comparative-effectiveness question is whether a surgery-based strategy provides an incremental benefit over definitive concurrent platinum–etoposide chemoradiotherapy followed by consolidation durvalumab in eligible patients with limited-stage SCLC. This comparison should use clearly defined eligibility criteria, standardized staging, and prespecified treatment protocols, while accounting for differences in tumor stage, operability, and baseline patient characteristics. Overall survival and event-free survival should be prioritized, with treatment-related morbidity, quality of life, treatment completion, and patterns of recurrence included as additional outcomes. Importantly, evidence supporting surgery in cT1–2N0M0 disease should not be interpreted as establishing superiority over the contemporary nonsurgical standard, because a direct comparison against this standard is lacking.

Feasibility and alternative evidence-generation strategies: Given the rarity of rigorously staged, surgically eligible early-stage SCLC, a conventional randomized trial directly comparing surgery-based multimodality treatment with definitive chemoradiotherapy followed by durvalumab may be difficult to recruit and sustain. This feasibility limitation should be acknowledged explicitly rather than assuming that a randomized comparison will necessarily be achievable. International cooperative-group collaboration and prospective registries with harmonized eligibility criteria, staging procedures, treatment definitions, and outcome collection could provide a feasible foundation for comparative research. Pragmatic or platform-based studies may also be considered where sufficient clinical equipoise, infrastructure, and recruitment capacity exist. In parallel, target-trial emulation using high-quality prospective and retrospective data could compare clearly specified treatment strategies by aligning eligibility criteria, treatment assignment, and follow-up from a common time zero, while addressing measured confounding, treatment-selection bias, and immortal-time bias. These observational approaches cannot fully eliminate residual confounding and should complement, rather than be presented as equivalent to, randomized evidence.

Interpretation of the proposed framework: Accordingly, a surgery-versus-surgery randomized trial should be presented as a study of treatment sequencing, whereas the incremental value of surgery requires a separate comparison against contemporary nonsurgical treatment or a carefully designed alternative comparative-effectiveness strategy. Both questions are clinically relevant, but they answer different questions and should have distinct objectives and interpretations.

What systemic treatment: Trial regimens should build on platinum-etoposide as the postoperative backbone for resected N0 disease, incorporating chemo-immunotherapy (as validated in extensive-stage IM power 133/CASPIAN); durvalumab consolidation as perioperative/adjuvant addition is not an established practice, representing at the moment a trial-only hypothesis [6].

What radiation: Trials must define RT strategy according to nodal outcome at resection—postoperative RT for unexpected pN1 disease should be considered selectively, whereas unexpected mediastinal pN2 disease warrants chemotherapy plus mediastinal radiotherapy [6].

What CNS strategy: Given that the benefit of PCI is unclear in patients treated with definitive therapy for pathologic stage I–IIA disease, trials should embed MRI brain surveillance as an alternative to PCI in this population, mirroring the design of the ongoing SWOG S1827/MAVERICK trial, which includes surgically resected patients and randomizes PCI versus MRI surveillance [6].

What MRD endpoint: Perioperative ctDNA offers a candidate intermediate endpoint, drawing on NSCLC experience where tumor-informed MRD assays predict recurrence-free survival with lead times of months over radiographic imaging and inform adjuvant therapy allocation; SCLC-specific ctDNA kinetics have similarly tracked treatment response and resistance, but tumor-informed MRD platforms and their prognostic thresholds remain unvalidated in resected SCLC and require dedicated study [42,43].

What molecular/translational endpoints: Trials should prospectively bank paired tumor and plasma at resection, induction, and relapse to enable serial transcriptomic subtyping, immune microenvironment profiling, and clonal evolution analysis, positioning surgical trials simultaneously as therapeutic and mechanistic platforms rather than efficacy studies alone [41].

What surgical approach: In SCLC, video-assisted thoracic surgery (VATS) and robotic-assisted thoracic surgery (RATS) should be viewed as techniques for delivering appropriately selected multimodality treatment—not as an expansion of surgical indications. When surgery is appropriate, VATS may reduce pain, improve early physical recovery, shorten hospitalization, and reduce readmissions compared with thoracotomy, potentially helping preserve the planned postoperative treatment sequence and minimizing delays to systemic therapy. RATS may offer additional dexterity, three-dimensional visualization, and precise nodal or hilar dissection, particularly in anatomically complex cases, although its benefits over VATS remain uncertain and are offset by greater cost, resource requirements, and a learning curve. Because these perioperative data are derived predominantly from non-small-cell lung cancer, whether minimally invasive surgery improves completion or outcomes of multimodality therapy specifically in SCLC remains unproven [45].

Furthermore, metabolomics may help identify metabolic subtypes, resistance mechanisms, and pharmacodynamic biomarkers—particularly for arginine, purine, glycolytic/lactate, and lipid pathways—but no metabolomic assay currently guides standard SCLC therapy. The most credible next step is prospective multi-omics trials linking serial blood and tumor metabolomics to treatment response and testing biomarker-selected combinations [44,46]. Molecular subtyping, circulating tumor DNA (ctDNA) and MRD assessment, immune biomarkers, and metabolomic profiling are promising research strategies in small-cell lung cancer (SCLC); however, their clinical utility in resectable disease remains investigational. At present, no validated molecular subtype, immune biomarker, metabolomic signature, or ctDNA/MRD threshold has been established to select patients for surgery, guide perioperative treatment, or determine adjuvant therapy [42,43,44].

Future metabolomics research in resectable SCLC should proceed in three stages. First, analytical validation should establish assay reproducibility, pre-analytical requirements, and consistency across laboratories and patient cohorts. Second, prospective observational studies should evaluate whether metabolomic signatures are associated with pathological response, recurrence, and survival, while accounting for disease stage and treatment exposure. Third, only signatures with adequate analytical and clinical validation should advance to prospective biomarker-guided interventional trials testing whether their use improves treatment selection and patient outcomes [47]. Until these steps are completed, metabolomic signatures should not be used to guide routine clinical decisions.

Collectively, this framework converts the current fragmented retrospective literature into a testable, biologically stratified prospective agenda for defining whether, and in whom, surgery adds value in the chemoimmunotherapy era (Table 2).

## 10. Conclusions

Modern staging identifies a small, distinct cT1–2N0 population in whom surgery remains guideline-supported but incompletely validated, while ADRIATIC defines the contemporary non-surgical benchmark for future comparative strategies.

The historical failure of surgery in unselected SCLC populations, established by older randomized trials comparing surgery with radiotherapy, does not translate into a blanket contraindication for the very early, node-negative disease now identifiable through modern staging, since those trials answered a different clinical question in a different staged population. NCCN guidelines already reflect this nuance by recommending lobectomy with mediastinal lymph node dissection for carefully staged cT1–2N0 disease, a recommendation grounded largely in retrospective, registry-based series that carry substantial selection bias and cannot substitute for prospective evidence.

What can be said with confidence is that surgery’s role can no longer be settled by extrapolating from decades-old randomized trials conducted before modern staging, molecular subtyping, or immunotherapy existed. Nor can it be settled by retrospective survival comparisons alone, given their inherent selection bias.

The rational path forward requires distinguishing between two complementary but fundamentally different research objectives. Randomized trials comparing upfront surgery with neoadjuvant chemoimmunotherapy followed by resection could clarify the optimal treatment sequence among patients selected for surgery. However, such comparisons cannot determine whether surgery provides an incremental benefit over contemporary nonsurgical treatment. Addressing this latter question requires comparison with definitive concurrent chemoradiotherapy followed by consolidation durvalumab in eligible patients with limited-stage SCLC.

A conventional randomized comparison may be difficult to conduct because rigorously staged, surgically eligible early-stage SCLC is rare. International cooperative-group collaboration, prospective registries, pragmatic or platform-based studies where feasible, and carefully designed target-trial emulation may therefore contribute to a more robust comparative evidence base. These approaches should incorporate standardized staging and treatment definitions, prespecified eligibility criteria, clinically meaningful survival outcomes, and appropriate methods to address treatment-selection bias and residual confounding. Molecular characterization and circulating tumor DNA-based minimal residual disease assessment may further refine patient stratification, although these biomarkers require prospective validation in resected SCLC.

Until comparative evidence becomes available, surgery should remain an option for carefully selected patients within a multidisciplinary, guideline-concordant framework, rather than being regarded as either an established superior strategy or an obsolete treatment. Ultimately, the central unresolved question is not simply how surgery should be integrated with systemic therapy, but whether and in which patients a surgery-based multimodality strategy offers a clinically meaningful advantage over contemporary nonsurgical treatment.

Until such trials are completed, the honest conclusion is that surgery’s place in SCLC management remains genuinely unresolved rather than either established or discredited. Securing funding for clinical trials addressing primarily surgical questions remains a significant challenge, particularly in comparison with trials evaluating novel systemic therapies, which are more readily supported by industry. Future studies in small-cell lung cancer should therefore explore alternative funding models, including institutional support and collaboration through cooperative clinical trial groups. In addition, carefully designed trials that incorporate surgery within a broader therapeutic strategy or evaluate a new indication may provide opportunities for collaboration with pharmaceutical partners while addressing important unmet surgical questions.

## Figures and Tables

**Table 1:** Present role of surgery and alternative treatments in SCLC.

Clinical Scenario	Present Role of Surgery	Preferred Operation	Required Systemic/RT Integration
Clinical stage I–IIA (cT1–2N0) after complete mediastinal staging; medically operable	Consider surgery as part of multimodality curative-intent care (minority of patients)	Lobectomy and systematic nodal dissection; aim R0; requires pathologic mediastinal staging	Adjuvant platinum– etoposide chemotherapy; PCI controversial/individualized
Incidental SCLC found after resection of a solitary pulmonary nodule	Complete staging and deliver multimodality adjuvant therapy; no role for “surgery alone”	If already resected: ensure margin/nodal assessment adequate; consider completion lobectomy case-by-case	Adjuvant platinum–etoposide chemotherapy; consider RT based on nodal status/margins
Pathologic N1 after resection (upstaging)	Surgery may still be “salvaged” as part of multimodality approach; prognosis worse than N0	R0 resection with adequate nodal dissection (already done)	Adjuvant chemotherapy mandatory; consideration of thoracic RT varies; evidence limited
Unexpected pathologic N2 and/or R1–R2 resection	Surgery not sufficient; requires escalation	-	Adjuvant chemotherapy + thoracic RT (preferably concurrent)
Clinical stage II–III (node-positive and/or locally advanced: N2–N3 or bulky mediastinal disease)	Generally not a surgical disease; standard is definitive concurrent chemo-radiation	-	Concurrent platinum–etoposide + thoracic RT
Extensive-stage (metastatic) SCLC	No role for curative resection	-	Systemic therapy ± palliative local RT
Medically inoperable early-stage (T1–2N0) SCLC	Surgery not feasible; use non-surgical local therapy	-	SABR or definitive RT + systemic therapy

SCLC, small-cell lung cancer; PCI, prophylactic cranial irradiation; RT, radiotherapy; SABR, Stereotactic Ablative Radiotherapy.

**Table 2:** Proposed Framework for Future Clinical Trials of Surgery in Small-Cell Lung Cancer.

Domain	Proposed Approach	Key Rationale/Basis
Which patients	Pathologically confirmed, invasively staged node-negative disease (cT1–2N0M0) Selected T3N0 or N1 in exploratory cohorts	NCCN-identified population most likely to benefit from resection; occult nodal disease must be excluded
Which molecular subtype (exploratory translational stratification)	Prospective ASCL1/NEUROD1/POU2F3/inflamed subtyping and TP53/RB1 status at enrollment	Test whether inflamed/antigen-presentation-competent phenotypes preferentially benefit from surgery-based immunotherapy strategies
Treatment sequencing within surgery-based strategies	In eligible cT1–2N0M0 patients, compare upfront lobectomy with systematic mediastinal lymph node dissection followed by adjuvant treatment versus neoadjuvant chemoimmunotherapy followed by resection.	Evaluates treatment sequencing among patients receiving surgery; cannot establish the incremental value of surgery over non-surgical treatment.
Comparative value of surgery versus contemporary nonsurgical treatment	Where feasible, compare a surgery-based multimodality strategy with definitive concurrent platinum–etoposide chemoradiotherapy followed by consolidation durvalumab in eligible patients with limited-stage SCLC.	Addresses whether surgery provides additional benefit over the contemporary nonsurgical benchmark; direct randomized evidence is currently lacking.
Feasibility and alternative study designs	Develop international prospective cooperative-group registries and consider pragmatic/platform-based studies and target-trial emulation using harmonized eligibility, staging, treatment, and outcome definitions.	Early-stage surgically eligible SCLC is rare; alternative comparative-effectiveness approaches may complement randomized evidence but remain vulnerable to residual confounding.
Systemic treatment	Perioperative or adjuvant immunotherapy after resection as investigational.	Builds on IMpower133/CASPIAN and ADRIATIC evidence.
Radiation	PORT for unexpected pN1 disease should be considered selectively, whereas unexpected mediastinal pN2 disease warrants chemotherapy plus mediastinal radiotherapy	Strategy defined by nodal outcome at resection
CNS strategy	MRI brain surveillance as an alternative to prophylactic cranial irradiation (PCI), embedded in trial design	Benefit of PCI unclear in pathologic stage I–IIA disease treated with definitive therapy; mirrors SWOG S1827/MAVERICK design
MRD endpoint	Perioperative ctDNA as candidate intermediate endpoint	NSCLC data show tumor-informed MRD predicts recurrence-free survival months ahead of imaging; SCLC ctDNA kinetics track response/resistance but remain unvalidated for resected disease
Molecular/translational endpoints	Serial banking of paired tumor and plasma at resection, induction, and relapse for transcriptomic subtyping, immune microenvironment profiling, clonal evolution analysis	Positions trials as mechanistic platforms, not just efficacy studies

PCI, prophylactic cranial irradiation; PORT, Postoperative radiotherapy.

## Data Availability

Data are available on request.

## Abbreviations

The following abbreviations are used in this manuscript:

SCLC	Small-Cell Lung Cancer
FDG PET	Fluoro-deoxy-glucose Positron Emission Tomography
CT	Computed Tomography
EBUS	Endobronchial Ultrasound
TBNA	Trans Bronchial Needle Biopsy
NCDB	National Cancer Data Base
SEER	Surveillance, Epidemiology, and End Results
IASLC	International Association for the Study of Lung Cancer
RCT	Randomized Controlled Trial
TMB	Tumor Mutational Burden
pCR	pathologic Complete Response
EFS	Event-free survival
MPR	Major Pathologic Response
NCCN	National Comprehensive Cancer Network
MRD	Minimal Residual Disease
PCI	Prophylactic Cranial Irradiation
PORT	Postoperative radiotherapy
ct DNA	Circulating tumor DNA
